# ‘Illuminating determinants of implementation of non-dispensing pharmacist services in home care: a qualitative interview study’

**DOI:** 10.1080/02813432.2023.2164840

**Published:** 2023-01-13

**Authors:** Karl-Erik Bø, Kjell H. Halvorsen, Torsten Risør, Elin C. Lehnbom

**Affiliations:** a Department of Pharmacy, Faculty of Health Sciences, UiT The Arctic University of Tromsø, Tromsø, Norway; b Department of Community Medicine, Faculty of Health Sciences, UiT The Arctic University of Norway, Tromsø, Norway; c Department of Public Health, Faculty of Health Sciences, Copenhagen University, Denmark; d Department of Health and Caring Sciences, Faculty of Health and Life Sciences, Linnaeus University, Kalmar, Sweden

**Keywords:** Clinical pharmacists, implementation science, patient care management, Norway, home care services, qualitative research

## Abstract

**Objectives:**

Medication errors are leading causes of hospitalization and death in western countries and WHO encourages health care providers to implement non-dispensing pharmacist services in primary care to improve medication work. However, these services struggle to provide any impact on clinical outcomes. We wanted to explore health care professionals’ views on medication work to illuminate determinants of the implementation success. The research was designed to inform and adapt implementation strategies for non-dispensing pharmacist services.

**Design:**

Semi-structured interview study with nine healthcare professionals.

**Setting:**

Four Norwegian home care wards.

**Subjects:**

Nine healthcare professionals working at different wards within one home care unit.

**Main outcome measures:**

Determinants of implementation outcomes.

**Results:**

Contextual determinants of the implementation process were mainly related to characteristics of the setting such as poorly designed information systems, work overload, and chaotic work environments. The identified barriers question the innovation’s appropriateness related to the setting’s needs but also provide possibilities for tailoring pharmacist services to local medication work issues. The observable positive effects and the perceived advantage of the pharmacist services are likely to facilitate the implementation process.

**Conclusion:**

Our study provided information on contextual elements that influence the implementation process of non-dispensing pharmacist services. Awareness of these factors can help develop strategies to help the organization succeed in in achieving program outcomes.

## Key points

- The results in this study illuminate barriers and facilitators to the implementation of pharmacist services in a home care setting.
- Existing medication work methods and poor information handover systems are likely to counteract outcomes of the pharmacist services and inflict unfavorable conditions for implementation.
- Healthcare professionals’ perception of increased medication work support and confidence in pharmacist skills suggest innovation acceptability and serve as indicators of implementation success. The identified barriers to improving medication work provide opportunities to develop tailored strategies to enhance the implementation of non-dispensing pharmacist services.

## Introduction

Medication errors are diverse and persistent matters within health care and the associated global cost is estimated to be $42 billion annually [1]. The erroneous use of medications is a leading cause of death and hospitalization in western countries and the prevention of avoidable harm is one of the most pressing issues in the field of patient safety. With an increasingly aging population, the incidence of co-morbidity and polypharmacy make patient care even more challenging as the risk of medication-related injury increases with the number of medications taken [2].

Adverse events caused by medication errors originate from a multitude of circumstances within both medical practice and medicines management and multifaceted approaches are needed to lower error rates [3]. Non-dispensing pharmacist interventions have proven to be beneficial in the reduction of adverse drug events within hospital settings [4] and pharmacist services are recognized as important measures to identify inappropriate medications and improve patient outcomes [1,5,6]. The integration of pharmacists into multidisciplinary healthcare teams has progressively gained interest but the success of improvement work requires a good fit between the new service and its context [7]. Moreover, the introduction of improvement services in organizations tends to be driven more by solutions and less by problems [8].

Adoption of new healthcare delivery models often requires an altering of existing practices and work methods. Moreover, introducing changes in healthcare is challenging and new, practice-improving services are often put in place without sufficient knowledge of factors that might emerge to influence the expected program outcomes [9]. The delivery context of an innovation and the organizational culture in which the innovation is applied encompass a dynamic network of factors and agents that both interact and act in parallel to each other. As such, opportunities for barriers are widespread and interconnected issues can cause unexpected chains of dependencies for seemingly isolated challenges. Furthermore, health care professionals are not passive recipients of innovation, but rather agents that are involved as important contributors to the process. Their actions, feelings, and attitudes towards the practice change add complexity to the innovation uptake [10].

The term *implementation* signifies a planned and systematic introduction of changes to the existing practices within a setting, often foregone by pre-implementation stages of contemplation, exploration, and decision-making. This comprehensive and active approach to integrating new services is associated with increased success in achieving the desired program outcomes [11]. A pivotal part of the implementation process in healthcare organizations is to gain knowledge on actual performance within a setting as this can reveal potential determinants of innovation success [12]. Moreover, understanding root causes, where they originate, how they are sustained, and whether they are susceptible to the chosen intervention can help develop and tailor services to local needs [13,14]. If the innovation should fail, this assessment can help explain to what degree the context and the implementation process rather than the innovation itself contributed to the failure.

In this study, the aim was to illuminate elements that influence the implementation of new improvement services within a primary care setting.

## Materials and methods

### The research team and reflexivity

This study was designed to inform on implementation strategies for non-dispensing pharmacist services and to alleviate potential barriers to improved medication work (innovation outcomes). It is part of a quality improvement project which seeks to develop and implement pharmacist services within a home care setting to create a positive change related to medication management. Stakeholders from the municipality health administration were actively involved in the planning of this study.

The research team included one female pharmacist (ECL), two male pharmacists (KEB and KHH), and one male general practitioner (TR). The first author (KEB) was a PhD-student with experience in managing community pharmacies. The rest of the research team (ECL, KHH, and TR) had backgrounds in health services research. All authors were familiar with the health care system and settings in which the research was performed.

### The research setting

Home health care services play an important role in preventing avoidable illness and hospitalization. Norwegian home care services are administered by municipalities and granted upon application. Medication work is a large part of this service and medication errors are frequently reported [15]. 

Data for this study were collected from four wards within one home care unit which, at the time of the study, was the only Norwegian home care unit having employed a full-time pharmacist. This specific approach to the improvement of medication work is novel in Norway, and to our knowledge, the home care setting in which we collected data was the first of its kind.

### Study design and data collection

The authors collected qualitative data to answer the research questions:

- What are the issues and challenges within medicines management?
- How do workflows influence the implementation process?
- How do the new services align with existing systems and practices?

To address these questions, the researchers participated in staff meetings (KEB), conducted observation visits with healthcare professionals (KEB), and interviewed healthcare professionals (KEB). The home care settings guidelines and procedures for medicines management were reviewed to better understand each part of the process.

Informants were recruited from within the specific setting of interest. Information regarding the research project was sent *via* e-mail to five ward managers who recruited participants from within their wards. The final composition of informants was a result of both purposive and convenience sampling strategies. Individuals were eligible to participate if they were actively involved in medication work. Moreover, the authors wanted to include health personnel from all five wards to capture potential differences between each site.

The development of the interview guide and the process of interviewing was inspired by a broader sense of phenomenological approach related to Elton Mayos method as described by Kvale, S., and Brinkmann, S [16]. Interview questions were deliberately broad at the start of the interview to avoid leading the participant in any direction, e.g. ‘can you tell me about your experiences with medication work?’. The interview guide is provided in Supplementary Appendix A.

Ten healthcare professionals aged 20–60 years signed a consent to participate in our study but one participant dropped out due to illness. As such, a total of nine interviews were conducted. The participants’ backgrounds were registered nurses: 4, ‘social educators (Norwegian: vernepleier’ [1]): 2, auxiliary nurses: 3, and they were not acquainted with the research team.

^‘^Social educator’ is a translation of the Norwegian ‘vernepleier’, a health care professional frequently involved in medication management [1].

Semi-structured interviews were carried out in April and May of 2021. Each interview lasted for 30–45 min and was audiotaped *via* Teams®. All interviews were carried out and transcribed verbatim by one researcher (KEB). Field notes were made after each interview. All participants received a ‘thank you’ voucher worth €50 upon completion of the interview.

### The on-site pharmacist and the non-dispensing pharmacist services

The role of pharmacists in Norway has traditionally been limited to medication dispensing activities within community pharmacies. In general, clinical pharmacist positions are rare, and few attempts have been made to integrate pharmacists into home care settings. Moreover, pharmacy residency programs do not exist in Norway and clinical training can only be obtained through work experience. In the setting we researched, the on-site pharmacist had clinical experience from a previous position.

The scope of the pharmacist services in the research setting was mostly determined by the pharmacist’s competencies but to some extent influenced by the healthcare team’s total workload. Standard operating procedures regulated parts of the medication work but the pharmacist was empowered to shape the new and innovative role within broader boundaries of improvement work.

Based on pharmacist observation visits and the information provided by the participants in this study we identified the non-dispensing services in the setting to target both patients (basic medication review, medicines reconciliation, education/training) and healthcare professionals (education/training, organization of medication work). The pharmacist’s working hours were Monday to Friday from 8 am to 4 pm.

### Analysis and theoretical framework

The movement of evidence-based practices into healthcare settings can be depicted as a continuum with two distinct processes on each extreme: ‘let it happen’ (passive diffusion) and ‘make it happen’ (implementation) [10]. The results in this study pertain to an ongoing uptake of improvement services in a home care setting and data were collected approximately 12 months into this process. Supplementary Table 1 provides a theoretical lens to the initiation and implementation process in organizations and highlights elements that have been associated with implementation success [8].

Several theories of implementation and complexity were reviewed to better understand how contextual factors might influence the diffusion process and program outcomes of improvement services in healthcare settings [17]. Determinants of implementation are often similarly arranged across different theories and frameworks and related to the following features of an organization: the innovation (a new service or practice), the inner setting (users of the innovation), characteristics of individuals, the outer setting (regulation, economic structures, policies), and the process [18]. In this study, our data relate to only two of these organizational features: the innovation, and the inner setting. Supplementary Table 2 provides an overview of how constructs from theoretical models can help assess determinants of implementation within these two characteristics of an organization.

The analysis was inspired by a thematic ‘bottom-up’ approach and consisted of the following steps: reading and re-reading transcripts, identification of meaning units relevant to our research questions, and condensing of these meaning units. Condensed meaning units were coded within each interview and the most relevant codes for our research questions were abstracted and clustered into themes. This process was iterative and carried out for each interview, transcripts were revisited several times during the research process. The results of the individual interviews were compared and ultimately combined in a cross-sectional analysis, and reviewed by all researchers (KEB, ECL, KHH, and TR). In the final stages of the analysis, domains and constructs from determinant frameworks guided the clustering of codes into themes.

### Ethics and consent

The research is approved by the Norwegian Center for Research Data (NSD). All participants signed an informed consent document. The document stated that health personnel had the right to withdraw from participation in the research at any time without providing any reason. This information was repeated to participants by the interviewer (KEB) upon completion of the interview.

## Results

The findings in this study are organized and related to two contextual domains that overlap between several determinant frameworks: the setting and the innovation [10].

### Characteristics of the setting

#### Workload, stress, and interruptions

Constructs like high workload, time pressure, and stress were reported across all interviews and participants experienced that they more often than not struggled to find time to complete medication management. Some estimated that they spent as much as 50% of their time on medication management and that they consequently had less time to nurse patients. Moreover, participants reported that they experienced time pressure to be the cause of frequent and repeating incidents of medication errors. Other utterances expressed concerns regarding the number of interruptions health personnel experienced during medication management. These interruptions frequently made health personnel unable to complete assignments during their shift:

Interruptions during medication work are very common: telephones ring constantly and people are knocking on the door to ask questions. (Healthcare provider/P1)

#### Information handover and communication

Participants reported that information handover from the hospital to the home care setting was troublesome and defective. Moreover, due to a lack of integrated data systems, the reconciliation of medication lists depended on health personnel’s ability to gather information by phone and electronic messages. Nurses reported that they had to cross-examine medication lists from the hospital with medication lists from the home care electronic system as well as with the general physician’s lists (GP) and lists from the community pharmacy. This work often required making phone calls to the hospital and the GP and some of the informants described that they spent several hours getting the necessary information by phone.

Poor communication was also related to the lack of proximity to a physician. Most informants experienced this as an obstacle to accessing information. Medication list discrepancies were often sought solved by sending electronic messages to the patient’s GP. One participant commented:

If we discover any medication discrepancies we send an electronic message to the GP to resolve the issue. They have a deadline to reply within 5 days and it might take a week before the error is corrected. (Healthcare provider/P3)

Informants reported that additional time was spent sending reminders to GPs urging them to respond to these messages. This situation was perceived as frustrating, and it made it difficult to solve pressing medication-related problems. Fridays were reported to be particularly difficult days to run into any medication-related issues. Any response from the GP would most likely be delayed over the weekend and into the beginning of the next week leaving health personnel and their patients in a situation of insecurity.

#### Work processes within the home care organization

The home care setting’s preferred way of dispensing medication was reported to be automated dose dispensing (ADD). A challenge with this system was that any changes to the patient’s medication regimen had to be reported within a deadline to be effectuated and included in the next medication interval. One account in our data described a situation in which a tablet was unintentionally omitted from the patient’s ADD for two months. The perception of this system as unreliable caused nurses to spend several hours every other week to make sure the pre-packed multi-doses complied with the latest version of the patient’s medication lists. As a result of low flexibility within the ADD system, nurses reported that they spent a fair amount of time re-packing pre-dispensed multi-doses. In case of a discrepancy, which occurred frequently, the pre-dispensed multi-dose was opened, medication removed or added before the multi-dose package or pouch was ultimately sealed and information updated:

A large part of the day is spent preparing medications. Even though most patients receive pre-dispensed medication discrepancies frequently make us re-dispense or manually dispense the medication, every week on several occasions. (Healthcare provider/P7)

#### Adherence to medication safeguards and guidelines

According to municipal standard guidelines for medication management, electronic documentation was mandatory for some medication work. A reported problem was that health care professionals in the home care setting did not comply with these guidelines and that steps in the process of administering medication to patients were documented on a vast amount of *ad-hoc* printed paper lists. Some participants described as many as six additional printed lists that were found to be used in parallel to the electronic list. This deviation from the standard electronic documentation made it difficult to keep track of medication errors and medication-related discrepancies:

We often forget to document administered medication during parts of the day when it is most hectic. This causes us to miss out on whether medication has been administered to the patient or not. (Healthcare provider/P2)

One participant described that medication errors often were reported by word of mouth and thus passed on to someone other than the person that discovered the error. The same participant pointed out that this way of reporting medication errors often led to a situation where errors were not documented at all. Moreover, interviewees highlighted medication errors at the point of administration as a pronounced challenge and that these kinds of errors re-occurred identically. One of the interviewed nurses described why these discrepancies occur:

Stress, insufficient staffing, and working in the automatic mode. We do not bother to read the text on the medication. Each dose is thoroughly labeled with the name of the patient, name of the medication, day, and time for dosage. You wouldn’t think it was possible to mess it up, but we do. (Healthcare provider/P7)

### Characteristics of the innovation

#### The pharmacist as a provider of new skills to the intradisciplinary team

The on-site pharmacist was reported to help increase medication knowledge among health personnel through training and education. Some participants reported that this aspect was one of the key features of a clinical pharmacist service and placed the pharmacist’s medicines knowledge above both nurses and GPs based on perceived pharmacological skills. Some participants emphasized the clinical importance of including the pharmacist as part of a multidisciplinary team to be able to handle increasingly complex patients with co-morbidities and polypharmacy. Moreover, the pharmacist’s ability to identify and solve medication-related issues, i.e. drug-drug interactions or other challenges related to pharmacology, was reported across several accounts. Reflecting on these issues, one participant expressed satisfaction with the collaboration with *community pharmacists* as well, even though the same participant found comfort in knowing that they now had access to a pharmacist located on-site.

#### The pharmacist as a target of medication-related inquiries

The scope of medication-related inquiries within the home care setting was reported to be vast and thus access to an on-site pharmacist was perceived as valuable and pertinent. The co-location was reported to be particularly important in situations where it was difficult to contact the patient’s GP. Moreover, healthcare workers’ ability to speak to the pharmacist face to face was perceived as extremely important across several accounts. As such, participants reported that inquiries previously directed towards physicians frequently were directed toward the pharmacist. Also, some participants described how they evaded medication inquiries and expressed a sense of relief that in the presence of a pharmacist, they were no longer the target for medication-related questions from colleagues. They justified these actions by assuming that the pharmacist was innately more capable of answering inquiries related to medication work. One participant commented:

Before we had access to the on-site pharmacist we had to deal with medication-related inquiries on our own. We had to spend time reading, searching online, and writing electronic messages to the GP. Now we use the pharmacist to manage all this work, and it saves us a lot of time. (Healthcare provider/P8)

And:

When someone approaches me with medication-related inquiries I reply: ‘Go talk to the pharmacist, she knows more about this topic than me’ (Healthcare provider/P6)

#### The pharmacist is only one piece in the medication improvement puzzle

Participants reported that the on-site pharmacist had reduced the total workload by actively adopting their work tasks. One example was the medicines reconciliation, which some of the participants associated with a degree of complexity. Despite each step being described in a standard operating procedure, they took for granted that the on-site pharmacist was better skilled to carry out this work.

Even though most participants expressed unanimous satisfaction with the pharmacist’s work few accounts articulated neither knowledge of, nor experience with, specific services. Moreover, some utterances were ambivalent about the impact of pharmacist services on medication work improvement; when probed on whether or not the participants would have chosen a pharmacist to improve medication work in the setting some accounts contained an explicit preference for health professions like nurses and physicians in place of the pharmacist. Nurses and physicians were perceived to be able to solve medication-related issues more effectively.

## Discussion

Barriers and facilitators related to the provision of non-pharmacist services have been reported for diverse healthcare organizations and specific interventions [19–21]. This study describes healthcare professionals’ views on perceived issues and challenges within medication work. It also presents participants’ perceptions of the advantage of the innovation, non-dispensing pharmacist services. As such, our data provide information that shed light on the context in which the new services are adopted. Supplementary Tables 1 and 2 provide models from implementation science to help conceptualize how these contextual elements might influence the implementation process and program outcomes.

### Stakeholders’ satisfaction with the innovation

Certain attributes of a new service tend to be favorable for the implementation process. One innovation feature that has been associated with successful adoption is captured in the theoretical construction of ‘relative advantage’, i.e. if involved stakeholders perceive a clear and visible advantage of the new services compared to what is currently used, implementation is more likely to succeed [10]. A similar implementation construct, ‘acceptability’, goes beyond general contentment and highlights that stakeholder satisfaction should be related to particular actions or specific services [22]. Moreover, an assessment of ‘acceptability’ should be based on the stakeholders’ knowledge of and experience with the services content.

The participants in our study articulated both specific knowledge of how the innovative services could facilitate medication work, and an appreciation of access to in-situ pharmaceutical knowledge. They portrayed the pharmacist as a versatile resource and unanimously pointed to optimizing the patients’ medication lists as an important component of the pharmacist intervention. Moreover, the benefits of pharmacist services were reported to be observable through social support, increased medication-related knowledge among health personnel, and improved benchmarking on quality indicators. Some accounts in our data compared aspects of medication work before and after the introduction of the innovation with specific examples of how several medication-related processes had improved. Medicines reconciliation was one such process.

### Is the situation intolerable without pharmacist services?

Implementation is more likely to succeed if health personnel within a setting is convinced that the innovation or adapted service is urgently needed [10,18]. The implementation construct ‘tension for change’ relates to how stakeholders perceive the current situation i.e. do they find the situation intolerable or sense an acute need to change? Many of the reported medication issues in our study appeared to be seemingly innovation-stabile, i.e. they did not necessarily pertain to the absence of pharmacological skills but rather to a stressful environment caused by staffing ratios.

High workloads were perceived as the root cause of medication work challenges and medication errors in the home care setting. Participants reported this core issue to trigger both stress and automaticity, i.e. performing work tasks independent of conscious control and attention. Insufficient staffing, an antecedent of increased workload, was another frequently reported issue and participants stated that staffing-induced stress inflicted an element of chaos on medication work and caused health personnel to pay less attention to procedures and systems safeguards. Low staffing levels were reported as persistent issues in the setting caused by both vacancies and sick leave. Reflecting on this chronic medication work condition, some participants expressed that they would prefer to replace the pharmacist with registered nurses or physicians who had the authority to resolve urgent medication-related issues. These accounts illuminate the legislative boundaries of Norwegian pharmacists’ and thus their limits of immediate impact on medication work: unlike physicians, they are not authorized to make any changes to medications, i.e. they cannot prescribe/deprescribe or alter doses. And unlike registered nurses, pharmacists do not have the authority to administer injections. Moreover, these data illuminate how latent and structural characteristics of the setting will influence medication work regardless of innovation delivery. Also, scant resources (e.g. deficient staffing) related to normal routine activities, work overload, and chaotic work environments are recognized to impose challenges to the implementation process [12,23,24].

### How do the new services fit with the setting?

Theoretical models like ‘appropriateness’ and ‘compatibility’ refer to how well an intervention fits with the setting’s latent systems and existing ways of working [18]. Alternatively, the term ‘Lack of a cohesive mission’, describes situations where actions and procedures within an organization conflict with the mission of the innovation [12]. It is important to emphasize that the assessment of ‘fit’ relates to the implantation process and, in our case, not to whether pharmacist services can improve medication work in the setting.

Accounts in our research described examples of everyday behaviors that counteracted the stated mission of the innovation, like neglecting to document medication errors and unwillingness to adhere to standard medication work procedures. These malpractices are found to characterize organizational cultures that grapple to improve quality [25] but they also make possible targets for tailored pharmacist services within the home care setting.

A root cause of medication-related issues was the lack of information technology infrastructure in the home care setting; poor information handover made it difficult to obtain an accurate and up-to-date medication list for patients. One medication-work malpractice related to this latent characteristic of the setting was illuminated through the situation in which missing information caused nurses to spend several hours *re-dispensing* machine-packed multi-dose medications every week. Automated dose dispensing (ADD) is a service targeted at people using multiple medications. Medicines are machine-packed into multi-dose units and thoroughly labeled according to the patients’ reconciled medication list. Even though there have been raised questions concerning the excellence of the ADD system over the last years, unit dosing is documented to improve rates of medication errors [4]. Moreover, the use of ADD is expected to reduce healthcare professionals’ workload, and decrease the medication cost [26]. The act of re-dispensing machine-packed medications is likely to counteract the advantages of ADD and increase the risk of medication error as additional steps are introduced into the medication work process [27]. Moreover, these actions will potentially thwart any preceding medication improvement services made by the pharmacist, e.g. medicines reconciliation [10,19–21,23–27].

### Validity of the findings

#### Data collection

The recruitment process was inspired by the concept of information power [28]. The authors had the opportunity to continue the data collection but chose to stop after ten planned interviews. This decision was based on the fact that our informants had firsthand information about the phenomenon of interest. Moreover, the phenomenon was familiar and well-known to both the participants and the researchers. The authors believe the number of participants was sufficient to provide answers to the research questions. Even though a higher number of informants might provide a stronger foundation for our results, data from a few individuals with first-hand knowledge can provide sufficient information on the core elements of medication work [29].

#### Thematizing the interview

Data were collected during an early stage of the innovation-decision process and the research setting encompassed a small number of health care professionals. The on-site pharmacist was well known to everyone in the setting and there were grand expectations of the effects of this novel initiative. Descriptions of the pharmacist as ‘mellow’ and ‘nice’ were frequent in most of the participants’ utterances. As three of the researchers are pharmacists, we took precautions to avoid the self-assumption that pharmacist services are the sole solution to improved medication safety. We applied an interview technique inspired by a neo-positivistic approach where the interview guide focused on the overarching phenomenon of medication work [30]. Questions were deliberately broad and open-ended to provide a more exploratory function. Utterances regarding the pharmacist and pharmacist services were probed for substance and clarity.

#### Limitations

As this research is part of a quality improvement project the results pertain only to the setting in which the informants were recruited. Participants were recruited from one home care unit only and there were fewer participants recruited from the more hectic wards within the unit. A possible reason is that they did not have the time to participate. Because of this, we might have missed out on information that could have provided us with additional important perspectives on medication work.

## Conclusion

This study illuminated several practice-related issues that are likely to influence the pharmacist’s ability to improve the medication work process in the setting. The most intolerable conditions reported by participants in this research, like staffing ratios and poor information handover, were latent and structural characteristics of the organization. These circumstances were reported to cause unfavorable environments for medication work resulting in medication errors, adverse events, and a suboptimal implementation climate. However, downstream issues of these root causes of medication error provide possible targets for tailored pharmacist interventions and might inform implementation strategies to better match the innovation with medication work challenges. Moreover, stakeholders’ clear perception of the pharmacist as better equipped to solve medication-related work increases the likelihood of successful implementation.

## Supplementary Material

Supplemental Material

Supplemental Material
