# Supplementary material for: ‘Illuminating determinants of implementation of non-dispensing pharmacist services in home care: a qualitative interview study’
Source: Scand J Prim Health Care. 2023 Jan 13;41(1):43–51. doi: 10.1080/02813432.2023.2164840 (PMC10088975; doi:10.1080/02813432.2023.2164840)
Supplement: Supplemental Material [file IPRI_A_2164840_SM0373.docx]

# Appendix B: Tables 1 and 2

Table 1: Simplified stages of the innovation process in organizations. Source: adapted from Rogers, 2003 Ch10 ^1^

| **Initiation:** (pre-) Contemplation and decision | |
| --- | --- |
| **Stage 1: Agenda setting**  The innovation process starts with a recognition of a need, problem, or issue. At this stage, answers often precede questions and sometimes knowledge of an innovation rather than the recognition of a problem launches the innovation process. | **Stage 2. Matching**  *“Effectively matching an innovation with an organization’s needs is key to whether the new idea can be sustained over time”* (Rogers, 2003, p. 391). The matching is pivotal to initiating the implementation stage and proceeding to the next steps in the process. |
| **Implementation:** Actions and maintenance | |
| **Stage 3. Redefining and restructuring**  Development of the innovation or services to accommodate the organization's needs. Generally, internally generated innovations tend to be more likely successfully implemented as the innovation more closely fits the organization's situation. | **Stage 4. Clarifying and routinizing**  The meaning of the innovation becomes clearer to the organization's members and services are put into more widespread use. Eventually, the innovation loses its separate identity and is incorporated into regular activities. |

Table 2: Determinant constructs related to the innovation and the inner setting of an organization.

| **The innovation (the new practice or service)** |
| --- |
| **Relative advantage:** Is the innovation better or more efficient than current practices in the setting? **^1-3^**  **Acceptability:** Is the specific content of the new services to the stakeholders' liking? ^4^  **Adaptability**: The innovation can be tailored and refined to fit local needs. ^3^  **Compatibility/appropriateness**: how do the innovation and its use align with existing work methods? ^1-4^ |
| **The inner setting (participants' workplace)** |
| **Tension for change**: do stakeholders perceive the current situation as intolerable? ^2,3^  **Compatibility/appropriateness**: how do the innovation and its use align with existing work methods? ^1-3^ |

1. Rogers EM, Rogers EM. *Diffusion of innovations*. 5th ed. Free Press; 2003.

2. GREENHALGH T, ROBERT G, MACFARLANE F, BATE P, KYRIAKIDOU O. Diffusion of Innovations in Service Organizations: Systematic Review and Recommendations. *The Milbank Quarterly*. 2004;82(4):581-629. doi:https://doi.org/10.1111/j.0887-378X.2004.00325.x

3. Damschroder LJ, Aron DC, Keith RE, Kirsh SR, Alexander JA, Lowery JC. Fostering implementation of health services research findings into practice: a consolidated framework for advancing implementation science. *Implementation Science*. 2009/08/07 2009;4(1):50. doi:10.1186/1748-5908-4-50

4. Proctor E, Silmere H, Raghavan R, et al. Outcomes for Implementation Research: Conceptual Distinctions, Measurement Challenges, and Research Agenda. *Administration and Policy in Mental Health and Mental Health Services Research*. 2011/03/01 2011;38(2):65-76. doi:10.1007/s10488-010-0319-7
