# Supplementary material for: ‘Illuminating determinants of implementation of non-dispensing pharmacist services in home care: a qualitative interview study’
Source: Scand J Prim Health Care. 2023 Jan 13;41(1):43–51. doi: 10.1080/02813432.2023.2164840 (PMC10088975; doi:10.1080/02813432.2023.2164840)
Supplement: Supplemental Material [file IPRI_A_2164840_SM0370.docx]

**Interview guide**

1. **Introduction/particpants background:**

- Can you tell me a little bit about yourself: age, profession, work experience.

**Research topics:**

**- preception of the medication management process**

**- preceptions of the on-site pharmacist services**

1. **Topic: medication management. Question:** **What are your experiences with everyday medication work?** **Follow up questions:**

- Can you describe medication-related tasks that you participate in?
- How do you perceive these tasks?
- Are you comfortable handling/participating in medication-related work?
- Are there different ways of handling medications between the different home care wards?
- In everyday work, how much time do you spend on medication-related tasks?
  - In your opinion: what works well? What does not work well?

1. Follow up questions related to challenges**:**

- What are the most pressing challenges?
- Are there different challenges related to different patient groups?
- Can you recall any episodes of medication error or discrepancies? Do you remember if the cause was identified?
- What are the routines for handling errors in your home care ward?

1. **Topic: the on-site pharmacist. Question: What are your experiences with the on-site pharmacist? Follow up questions:**

- How often have you had the opportunity to collaborate with the pharmacist?
- In your opinion, which skills separate a pharmacist from a nurse?
- What kind of services does the pharmacist provide?
- What are the pharmacist's most important contributions to medication management?

1. General probes:

- **Can you elaborate on this topic?**
- **Can you give some examples?**
